# Fluorescent Labeling and Imaging of IL-22 mRNA-Loaded Lipid Nanoparticles

**DOI:** 10.21769/BioProtoc.4994

**Published:** 2024-05-20

**Authors:** Rabeya Jafrin Mow, Anand Srinivasan, Eunice Bolay, Didier Merlin, Chunhua Yang

**Affiliations:** 1 Institute for Biomedical Sciences, Center for Diagnostics and Therapeutics, Digestive Disease Research Group, Georgia State University, Atlanta, GA, USA; 2 Yale University, New Haven, CT, USA; 3 Department of Chemistry, College of Arts & Sciences, Georgia State University, Atlanta, GA, USA; 4 Atlanta Veterans Affairs Medical Center, Decatur, GA, USA

**Keywords:** Oral gene delivery, Biodistribution, Gastrointestinal tract, Ulcerative colitis

## Abstract

Lipid nanoparticle (LNP)-based drug delivery systems (DDSs) are widely recognized for their ability to enhance efficient and precise delivery of therapeutic agents, including nucleic acids like DNA and mRNA. Despite this acknowledgment, there is a notable knowledge gap regarding the systemic biodistribution and organ accumulation of these nanoparticles. The ability to track LNPs in vivo is crucial for understanding their fate within biological systems. Fluorescent labeling of LNPs facilitates real-time tracking, quantification, and visualization of their behavior within biological systems, providing valuable insights into biodistribution, cellular uptake, and the optimization of drug delivery strategies. Our prior research established reversely engineered LNPs as an exceptional mRNA delivery platform for treating ulcerative colitis. This study presents a detailed protocol for labeling interleukin-22 (IL-22) mRNA-loaded LNPs, their oral administration to mice, and visualization of DiR-labeled LNPs biodistribution in the gastrointestinal tract using IVIS spectrum. This fluorescence-based approach will assist researchers in gaining a dynamic understanding of nanoparticle fate in other models of interest.

Key features

• This protocol is developed to assess the delivery of IL-22 mRNA to ulcerative colitis sites using lipid nanoparticles.

• This protocol uses fluorescent DiR dye for imaging of IL-22 mRNA-loaded lipid nanoparticles in the gastrointestinal tract of mice.

• This protocol employs the IVIS spectrum for imaging.

## Background

In ulcerative colitis, interleukin-22 (IL-22) plays a crucial role by promoting mucosal healing and regulating the inflammatory response. Lipid nanoparticles (LNPs) offer a targeted delivery platform for IL-22 in this context, effectively harnessing the cytokine's therapeutic potential to address mucosal healing and inflammation precisely at the site of injury [1]. Despite the increasing interest in utilizing LNPs for targeted delivery of therapeutic agents in ulcerative colitis, our understanding of their in vivo behavior is limited, hindering the clinical translation of LNP-based therapies. The tracking of LNPs in vivo can provide crucial insights into their biodistribution, migration abilities, and mechanism of action [2]. Therefore, the development of efficient and sensitive techniques for labeling LNPs is highly desired.

To date, several methods have been developed to unravel the in vivo dynamics of LNPs. Notably, the use of fluorescent dyes to label LNPs stands out as an effective approach for confirming successful therapeutic delivery. This highly sensitive and selective technique enables real-time monitoring and visualization of nanoparticle behavior and distribution in biological systems. However, a potential limitation of fluorescent labeling is the risk of dye leakage from nanoparticles in vivo, resulting in diminished brightness over time and the development of a background signal that may hinder accurate nanoparticle localization [3].

In a prior study, we engineered LNPs loaded with IL-22 mRNA for treating ulcerative colitis, evaluating the biodistribution of DiR-labeled IL-22/LNP [4]. In this protocol, we will describe the detailed process of labeling and imaging IL-22/LNP in the gastrointestinal (GI) tract using a fluorescent dye via the IVIS spectrum. In our previously published study [4], LNPs loaded with mRNA as described in this protocol displayed a distinct signal in the targeted organ (colon) and exhibited therapeutic efficacy.

## Materials and reagents

**Biological materials**

1. C57BL/6J mice (Jackson Laboratory, female, 6–7 weeks of age)

**Reagents**

1. Curved feeding needles (Kent Scientific, catalog number: FNC-20-1.5-2)
2. 1,1'-Dioctadecyl-3,3,3',3'-Tetramethylindotricarbocyanine Iodide (DiR') (Thermo Scientific, Invitrogen^TM^, catalog number: D12731)
3. Dimethyl sulfoxide (DMSO) (Fisher Scientific, catalog number: BP231-100)
4. Phosphate-buffered saline (PBS) (Corning, catalog number: 21-040-CV)

**Laboratory supplies**

1. 15 mL conical centrifuge tube (Thermo Scientific, Nunc^TM^ 15 mL, catalog number: 339650)
2. Amicon^®^ Ultra 15 mL centrifugal filter (Millipore, Ultracel^®^-100k, catalog number: UFC910024)
3. 5 mL Eppendorf tube (Eppendorf, catalog number: 0030119401)
4. 1 mL syringe PP/PE without needle (Sigma-Aldrich, catalog number: Z683531-100A)
5. Petri dish (CELLTREAT, catalog number: 229620)

## Equipment

1. Micropipettes, 10–100 µL (Eppendorf, catalog number: 13-684-251)
2. Orbital shaker (CORNING, model: LSE^TM^)
3. Centrifuge (Thermo Scientific, model: SORVALL ST-16R)
4. In vivo imaging system (PerkinElmer, model: IVIS Spectrum CT)

## Software and datasets

1. Living Image software (PerkinElmer, IVIS^®^ version: 4.7.4)

## Procedure

- **Fluorescent labeling of IL-22 mRNA-loaded LNPs**
  1. Prepare 5 mL of IL-22 mRNA-loaded LNPs in a 15 mL conical centrifuge tube following the previously published protocol [5].
  2. Dissolve 5 mg of DiR' powder in 1 mL of DMSO to make a DiR'/DMSO solution at a concentration of 5 mg/mL.
  3. Add 10 µL of DiR'/DMSO solution to 5 mL of IL-22/LNPs in a 15 mL conical centrifuge tube (Figure 1).
    **Figure 1.**
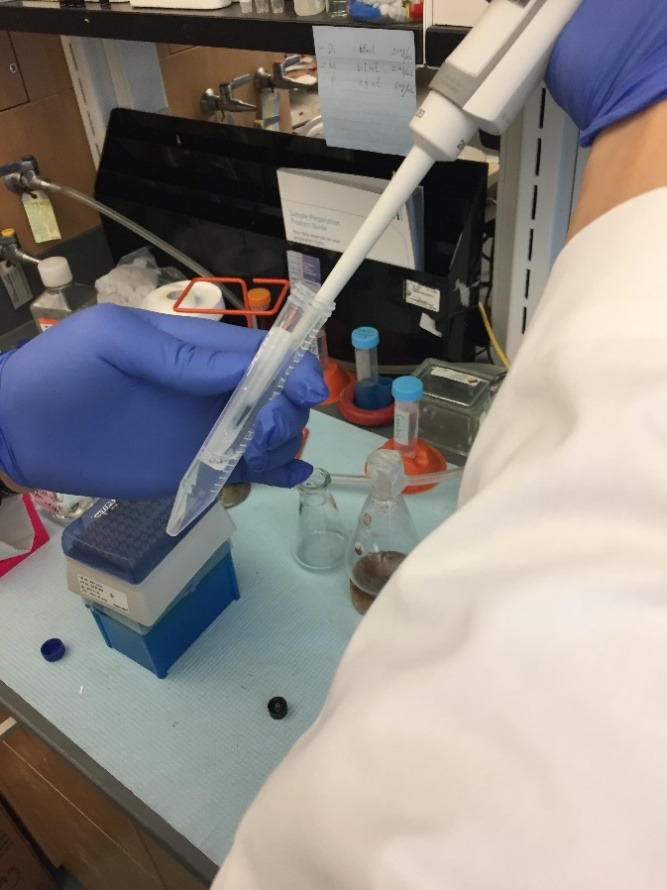
Spiking a 10 µL DiR' solution into the IL-22/LNPs suspension
  4. Cover the tube with aluminum foil and incubate the suspension mix at room temperature (RT) for 15 min (Figure 2, left) shaking at 100 rpm on an orbital shaker.
    **Figure 2.**
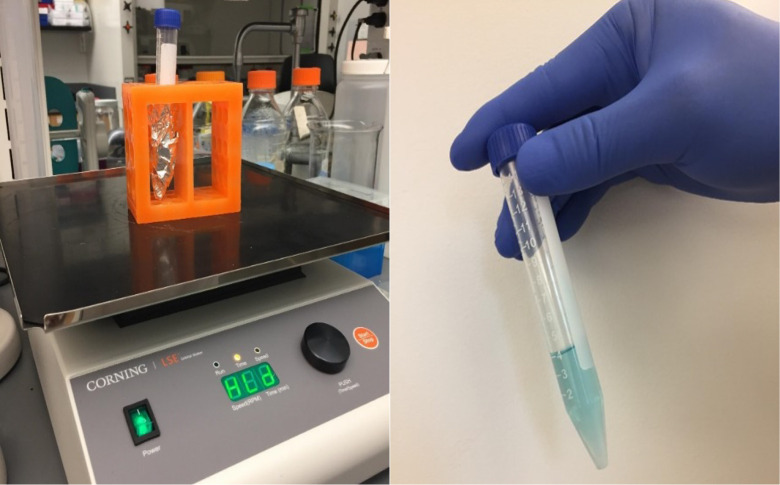
Incubating the lipid nanoparticles (LNPs) DiR' mix on an orbital shaker
  5. Transfer the LNP suspension to 15 mL centrifugal filters, each containing 2.5 mL of LNPs (Figure 3).
    **Figure 3.**
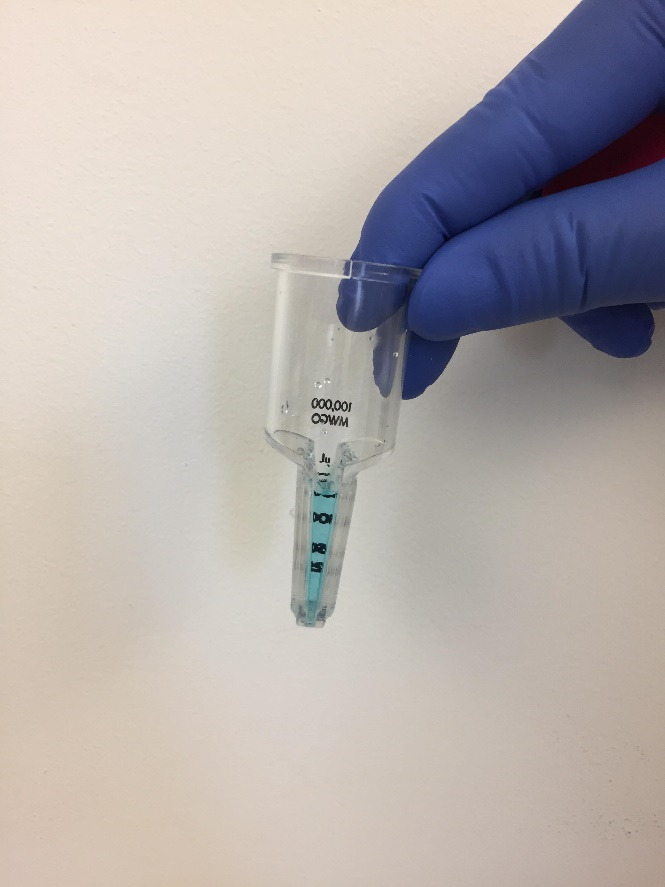
Transfer the lipid nanoparticles (LNP) suspension to a centrifugal filter
  6. Centrifuge at 4,696× *g* for 10 min at 10 °C.
  7. Take out the filter (Figure 4).
    **Figure 4.**
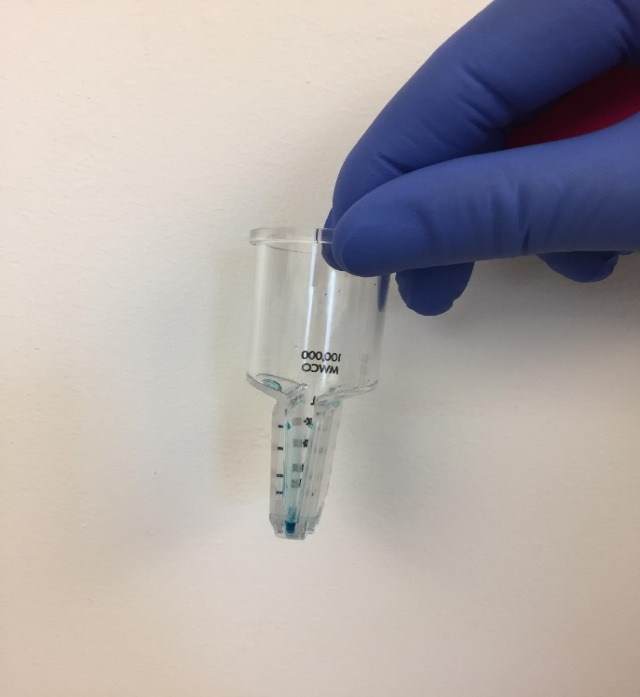
DiR'-labeled lipid nanoparticles (LNPs) in the filter after centrifugation
  8. Add 1 mL of PBS to each filter and reconstitute the suspension by repeated pipetting (> 100 times). Then, transfer the suspension to a 5 mL Eppendorf tube.
- **Oral gavaging of DiR'-labeled IL-22/LNPs**
  1. Take healthy mice and divide them into two groups (*Control* group and *Treated* group).
  2. Fast the mice for 4 h before gavage.
  3. Fill DiR'-labeled IL-22/LNPs suspension into a 1 mL syringe equipped with an animal feeding needle and remove all air bubbles (Figure 5).
    **Figure 5.**
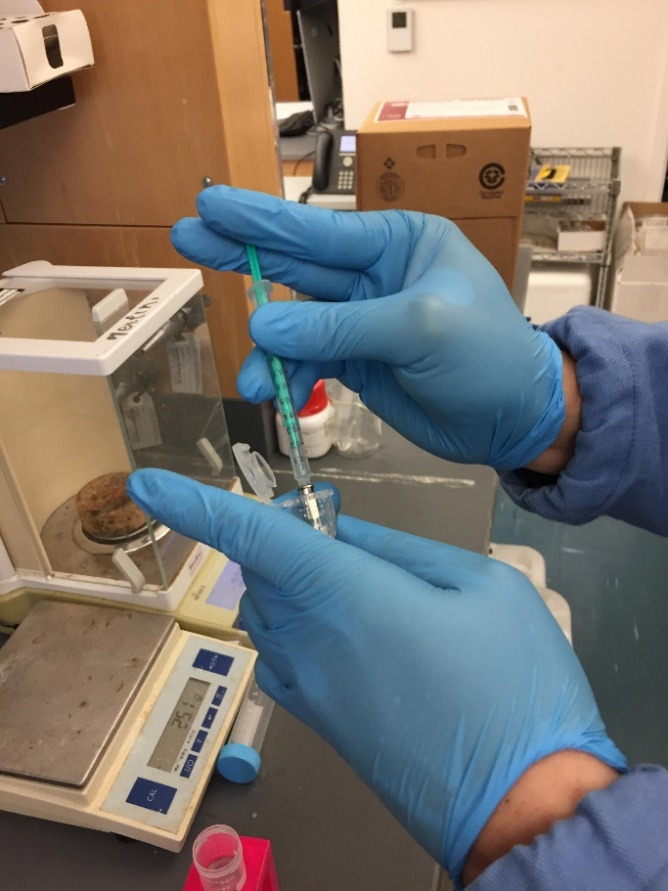
Filling of DiR'-labeled IL-22/LNPs suspension into syringe
  4. Restrain each mouse by manually grasping it and carefully insert the feeding needle into its mouth and esophagus to administer 200 µL of DiR'-labeled IL-22/LNPs suspension directly into the stomach of the treated group. To the control group, administer 200 µL of free LNPs suspension (without labeling) (Figure 6).
    **Figure 6.**
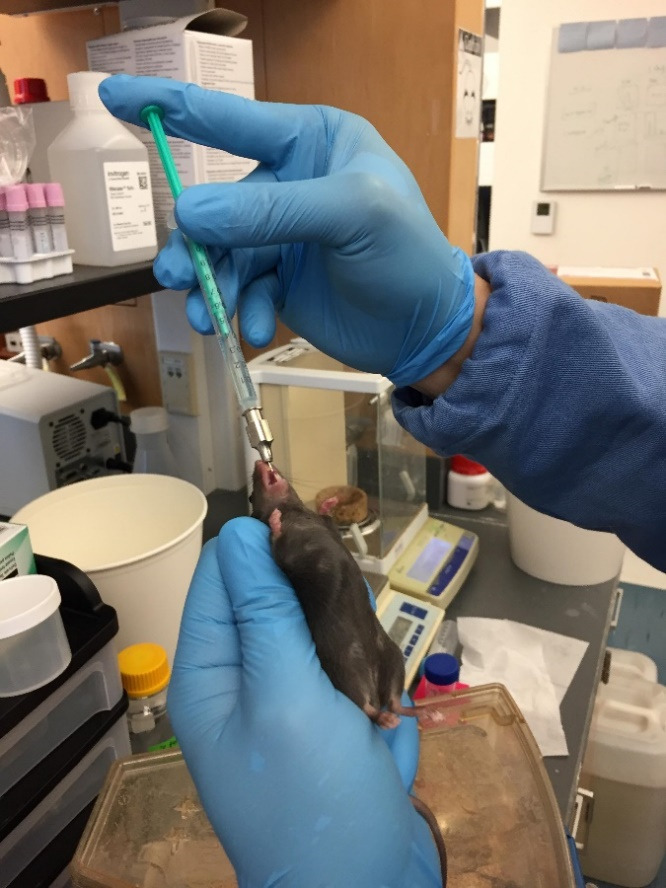
Gavaging of DiR'-labeled IL-22/LNPs suspension to mouse
*Note: If any resistance is experienced, it may indicate incorrect positioning of the feeding needle; in such cases, retract the feeding needle and reposition it.*
  5. After gavage, fast the mice for 1 h.
  6. Euthanize mice 24 h after being gavaged with mRNA-loaded LNPs.
  7. Dissect the mice and collect their GI tract.
- **Imaging of DiR-labeled IL-22/LNPs biodistribution in mouse GI tract**
  1. Turn on the IVIS Spectrum CT instrument 45 min prior to the test.
  2. Start Living Image software, click the initializing button, and wait until the temperature button turns green (Figure 7; CCD camera reaches -90 °C).
  3. Place the GI tract (with and without DiR-labeled IL-22/LNPs) in the imaging chamber.
  4. Set the imaging mode to fluorescence, excitation filter to 745 nm, and emission filter to 800 nm in the IVIS acquisition control panel (Figure 7).
  5. Take imaging by selecting *Acquire* in the IVIS acquisition control panel and save the acquired pictures to the appropriate path (Figure 8).
    **Figure 7.**
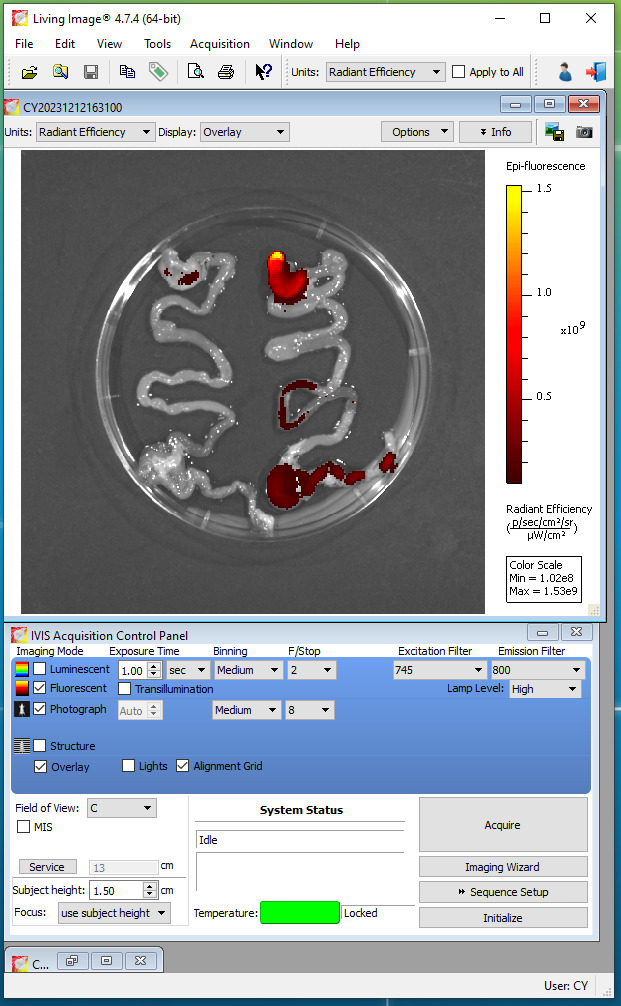
Parameter for acquiring imaging of fluorescently labeled IL-22/LNPs
    **Figure 8.**
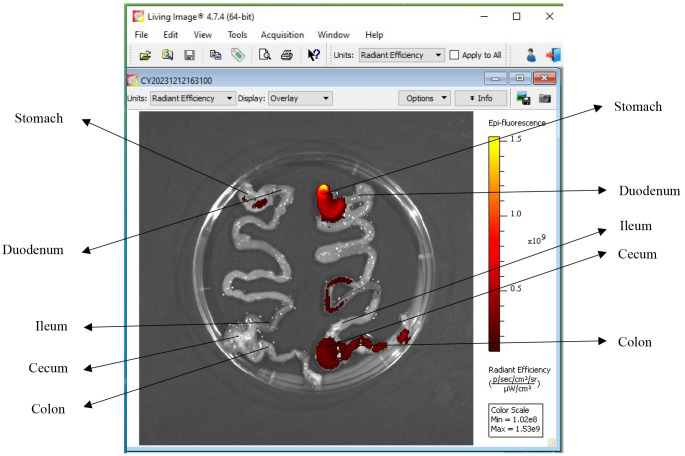
Living Image software. Left side: GI tract of the control mouse without IL-22/LNPs treatment. Right side: DiR-labeled IL-22/LNPs deposition in the gastrointestinal (GI) tract of the treated mouse.

## Validation of protocol

- Sung et al. [4]. Oral delivery of IL-22 mRNA-loaded lipid nanoparticles targeting the injured intestinal mucosa: A novel therapeutic solution to treat ulcerative colitis. Biomaterials (Supplementary Figure 10, panel A).
